# Atlanta as a Medical Center

**Published:** 1886-02-20

**Authors:** 


### ATLANTA AS A MEDICAL CENTER.

Atlanta is emphatically a medical center. It completely fulfills all the conditions appertaining thereto. Strangers or other persons of means, overtaken by disease and wishing to avail themselves of the best appointments for proper care, can find refuge without the sacrifice of any of their liberties. They can, not only obtain appropriate hospital and private rooms, and trained nurses, but they can secure such skillful and distinguished medical services and attention as will compare favorably with any attainable in this broad Union. Atlanta counts several medical schools, surgical institutions, hospitals, and infirmaries ; prominent amongst them stands the Southern Medical College, to which the Ivy-street Hospital is attached. The wisdom and propriety which prompted the foundation of this great Institution have been demonstrated by the steady growth and development of the College ; by the great popularity it has acquired at home and abroad, and by the assembling of classes of students characterized by intelligence, good deportment and earnest application to study. Hundreds of its graduates are scattered through this Continent. Not a few have attained considerable distinction, and the vast majority are successful and prosperous in their career.

The success of this College has been almost unprecedented. Though established in 1878, it has, in a few years, attracted larger classes than many of the oldest Medical Colleges in the United States. The paramount feature of this Institution is the *union of Clinical and Didactic teaching*. This constitutes its tower of strength; and the importance attached by the medical profession to this plan of instruction has been fully appreciated by the Faculty, and from the very inception they have set before themselves and their students a high standard of excellence, which has been raised each year, and they are determined to keep the curriculum abreast of the best Medical institutions in this country.

Atlanta now claims a population of 56,000. This affords a large supply of material for clinical instruction, and secures practical teaching, thoroughness, and completeness of medical investigation and demonstration.

